# Efficacy and Safety of Stem Cell Therapy in Advanced Heart Failure Patients: A Systematic Review with a Meta-analysis of Recent Trials Between 2017 and 2019

**DOI:** 10.7759/cureus.5585

**Published:** 2019-09-06

**Authors:** Joseph S Jayaraj, Rajesh Naidu Janapala, Aisha Qaseem, Norina Usman, Nida Fathima, Tooba Kashif, Vineeth K Reddy, Sanjiv Bakshi

**Affiliations:** 1 Internal Medicine, Icahn School of Medicine at Mount Sinai, Queens Hospital Center, New York, USA; 2 Internal Medicine, Emory Johns Creek Hospital, Georgia, USA; 3 Internal Medicine, Veterans Affairs Palo Alto Health Care System - Stanford University School of Medicine, Palo Alto, USA; 4 Internal Medicine, Sri Siddhartha Medical College, Tumkur, IND; 5 Cardiology, Heart and Vascular Institute, Dearborn, USA; 6 Internal Medicine, Sri Ramachandra Institute of Higher Education and Research, Chennai, IND; 7 Cardiology, Icahn School of Medicine at Mount Sinai , Queens Hospital Center, New York, USA

**Keywords:** heart failure, cardiomyopathies, chronic ischemic heart disease, autologous adult bone-marrow-derived stem cells, mesenchymal stem cells, stem cell therapy, human induced pluripotent stem cells, heart failure therapy, safety, efficacy

## Abstract

Objective

The effects of stem cell therapy in patients with advanced heart failure is an ongoing debate. This study aimed to assess the effectiveness and safety of stem cell therapy plus the standard of care as compared to the placebo plus the standard of care in advanced heart failure patients.

Methods

A comprehensive keyword search of PubMed between 2017 and 2019 was performed to extract trials conducted with stem cell therapy controlled with placebo in advanced heart failure. We included randomized controlled trials (RCTs) with data on safety and efficacy in patients with advanced heart failure after stem cell transplantation.

Results

Six RCTs, consisting of 569 patients, were selected. Three-hundred sixty-seven (367) out of 369 participants from the eligible four out of six RCTs were included for efficacy analysis, as we lost two patients from the final analysis due to early death. Five-hundred twenty-six (526) out of 527 participants from the eligible five out of six RCTs were included for safety analysis, as we lost one patient from the final analysis for not being able to receive the intervention. Stem cell transplantation significantly improved left ventricular ejection fraction (LVEF) by 4.58% (95% CI: 3.73-5.43%; p = 0.00001), improved left ventricular end-systolic volume (LVESV) by -5.18 ml (95% CI: -9.74 to -0.63 ml; p =0.03), and there was no difference in the risk of all-cause mortality (OR 0.97; 95% CI: 0.52 to 1.78%; p = 0.91). The above results correlate with the previous meta-analysis data conducted in 2016.

Conclusions

This meta-analysis provided the cumulative efficacy and safety results of stem cell transplantation in advanced heart failure based on recent RCTs. The above results suggest that stem cell therapy was associated with a moderate improvement in LVEF, and the safety analysis indicates no increased risk of mortality in patients with advanced heart failure. This meta-analysis recommends conducting more RCTs comparing stem cell transplantation and placebo with a larger patient population and longer follow-up.

## Introduction

Clinical vignette

"A 68- year old male presented to the hospital with chest pain in September 2008 and he underwent an Electrocardiogram (EKG) which showed a finding of ST-Elevation Myocardial Infarction (STEMI) and later the finding got confirmed by positive cardiac biomarkers, and thus diagnosed with Myocardial Infarction (MI). Following which the patient was admitted and treated according to the standard of care available in the hospital. The treatment provided was thrombolytic therapy as per the American College of Cardiology/American Heart Association (ACC/AHA) guidelines, following thrombolysis, the patient developed complications of ventricular fibrillation, heart failure, and pulmonary infection. The patient underwent angiography and Percutaneous Coronary Intervention (PCI) with stent placement in the anterior descending branch. Status Post PCI, the patient continued to be symptomatic despite the standard of care provided (aspirin, clopidogrel, atorvastatin, Angiotensin-Converting Enzyme Inhibitor (ACEI), Low Molecular Weight (LMW) heparin) for MI. Later the patient's heart function was classified as stage iii-iv according to the ACC heart failure classification and was treated with furosemide and spironolactone. After nine months, the patient underwent an alternate intervention with allogeneic umbilical Cord Blood Mononuclear Cells (CB-MNC's). The patient underwent an intravenous infusion with 2.0 × 107 allogeneic CB-MNCs, and before every injection, 5mg Dexamethasone and 25mg Phenergan were added to prevent any immune-related response in the patient. The dosing schedule was four injections/week, and the patient was kept under monitoring for any adverse effects, especially during cell transplantation. Posttransplantation at 24-48-hour point, a 24-hour EKG was monitored. After 3 months of intervention, the results of the study were (Left Ventricular Ejection Fraction) LVEF was significantly improved by 20%, the initial improvement was consistent even after six years, the scar size reduced by 11% post CB-MNC treatment which was measured by comparison of Single-Photon Emission Computed Tomography (SPECT) analysis done immediately after the MI episode and at 3 months post CB-MNC's transplantation, but compared to three months post CB-MNC transplantation, the scar size at 1 year post CB-MNC treatment remained the same. The High N-Terminal Prohormone of Brain Natriuretic Peptide (NT-proBNP) levels after nine months of MI normalized after CB-MNC treatment; the Patients activity improved significantly after CB-MNC treatment, and during the follow-up period the patient was able to walk 400m in a 6 min walk test. Overall, after treatment with CB-MNC's, LVEF improvement and scar size reduction was noticed, but there was a negative correlation with results of 6 min walk test and NT-proBNP levels after six months of therapy" [1].

Heart failure falls under one of the leading causes of mortality and morbidity in the USA. Modern science is expanding knowledge to help the medical fraternity explore more about the possible causes, mechanisms, and treatment options for heart failure. Heart failure is the result of inadequate pumping of blood and oxygen to other organs of the body. The United States has 5.7-million adults with heart failure [2]. In 2009, one in every nine deaths was due to heart failure [2]. Fifty percent of heart failure patients die within the next five years [2]. The USA spends $30.7 billion each year on heart failure [3], which includes health care service expenditure, heart failure medications, and missed days of work. Conditions causing damage to the heart also increase heart failure risk such as acute coronary disease (the most common type of heart disease), myocardial infarction, hypertension, and diabetes. The risk for heart failure increases with bad lifestyle practices, especially if already affected by one of the above diseases, which includes cigarette smoking, lack of physical activity, overweight, and dietary habits such as consuming food with highly saturated fats/cholesterol and high intake of sodium. Common symptoms of heart failure include dyspnea, orthopnea, pedal edema, and malaise. Early diagnosis and treatment can improve the prognosis of heart failure. Treatment usually involves medications, good dietary habits, and increasing physical activity. In heart failure, the standard of care is often limited. Current treatment options are medical therapy, implantable devices, and heart transplantation. The only definitive treatment of these is transplantation, which is limited by cost, eligibility, and availability. There are also implantable devices that are limited by high rates of complication and cost. 

The ongoing increase in heart failure prevalence urges new treatment options. The heart has no intrinsic regenerative capacity. Regenerative medicine has been extensively investigated to find a solution to this problem. There is excellent clinical enthusiasm for stem cell therapy as evidenced by the results in the above clinical vignette, and primarily investigated as a treatment option for heart failure with reduced function for over a decade, Experimental studies have reported an improvement in heart function and the repair of damaged heart tissue through various mechanisms such as transdifferentiation, cell fusion, and paracrine modulation [4-5]. Overall safety, benefit, best cell source, dosage, and route of administration remain unsettled. So, this article attempts to explore the safety and efficacy profile of various stem cell therapies and to add stem cell therapy as an extension or at least as an adjuvant to current treatment strategies for heart failure. We have attempted a systematic exploration of the recent literature from 2017 to 2019. Studying stem cell therapy in-depth and understanding the possible mechanism of action, dosage, and types of stem cells that we can use in heart failure treatment. The study about stem cells will not only help doctors and scientists to enhance their knowledge base, but it will also help save millions of lives all over the world.

This meta-analysis will critically explore the various randomized controlled trials (RCT) to find an answer to the safety and efficacy profile of stem cell therapy in advanced heart failure patients. In the end, we will recommend more studies to increase and fill the knowledge gap that is currently unknown.

## Materials and methods

In order to study, in detail, the safety and efficacy profile of stem cell therapy in heart failure, a comprehensive review of published literature was conducted via a PubMed search. Articles included were those relevant to the theme of heart failure/cardiomyopathy, stem cell therapy, its safety, and efficacy. The search terms were independently developed by two reviewers and then combined to perform a comprehensive search of relevant literature through the PubMed search engine and were screened according to the following criteria.

Inclusion/exclusion criteria

The inclusion/exclusion criteria used for our analyses are as follows: 

1. Studies (RCTs) that explicitly mentioned the terms heart failure (and synonyms) and stem cell therapy in the title, keywords, or abstract were included, whereas those that did not were excluded

2. Only peer-reviewed articles were included; all gray literature was excluded.

3. Irrelevant articles, ongoing trials, and duplicated RCTs were excluded.

4. We included the studies published from 2017 to 2019.

5. All articles in the English language were selected. Articles in languages other than English were selected only if an English translation was available.

6. The criteria of data selection strictly included articles focusing on the safety and efficacy profile of stem cell therapy in heart failure.

7. Articles only with human data were included, adults above 19 yrs of age were included, and ages below 19 and articles with only animal data were excluded.

8. The selection was mainly focused on RCTs; bibliographies from the reference lists of the published articles with the same focus were also selected.

Data extraction and quality assessment

Quality assessment and data extraction were done in duplicate by two authors independently. A detailed study of the various trials, including patient characteristics, treatment, outcomes, adverse events, and quality, was performed. One of the main outcomes was the left ventricular ejection fraction (LVEF). Out of the several methods of LVEF assessment, echocardiography (ECHO) was selected, as ECHO was a common method among most trials. All data available about serious cardiovascular events during follow-up were extracted. Twelve months follow-up data were collected among all trials. Quality appraisal for a meta-analysis was done with AMSTAR (Appraisal tool for systematic reviews of randomized and observational studies) checklist [6]. All the RCTs were assessed using the latest revised Cochrane Risk-of-Bias (RoB) tool for randomized trials (RoB 2) [7], which included various domains such as randomization, blinding and others as listed in the RoB 2 tool. All other studies were assessed for their quality according to their specific study type using a critical appraisal checklist from the Joanna Briggs Institute (JBI) [8-11]. Each questionnaire had 10-11 questions. Each question was given one point. A study scoring five or fewer points was considered as having a high risk of bias.

Statistical analysis

All outcomes were analyzed using the RevMan software (Review Manager (RevMan) computer program, version 5.3, Copenhagen: The Nordic Cochrane Centre, The Cochrane Collaboration, 2014). Statistical heterogeneity for the outcomes of interest was quantified using the I^2^ statistic, which gives information regarding the percentage of total variation due to heterogeneity rather than chance among the studies. As a means to calculate the efficacy, results were summarized as the weighted mean difference (WMD), with 95% confidence intervals (CI) using the random or fixed effects model as per study-to-study variability. The random-effects model was used when study-to-study variability by chance alone exceeded expectations. All p-values were derived from two-tailed statistical tests. Sensitivity analysis was performed by evaluating the effect of the individual study on the overall effect by excluding the LVEF result of one RCT at a time and computing a meta-analysis for the remaining studies, which assessed the change in the overall effect caused by the exclusion of any particular study.

## Results

Search results

**Figure 1:**
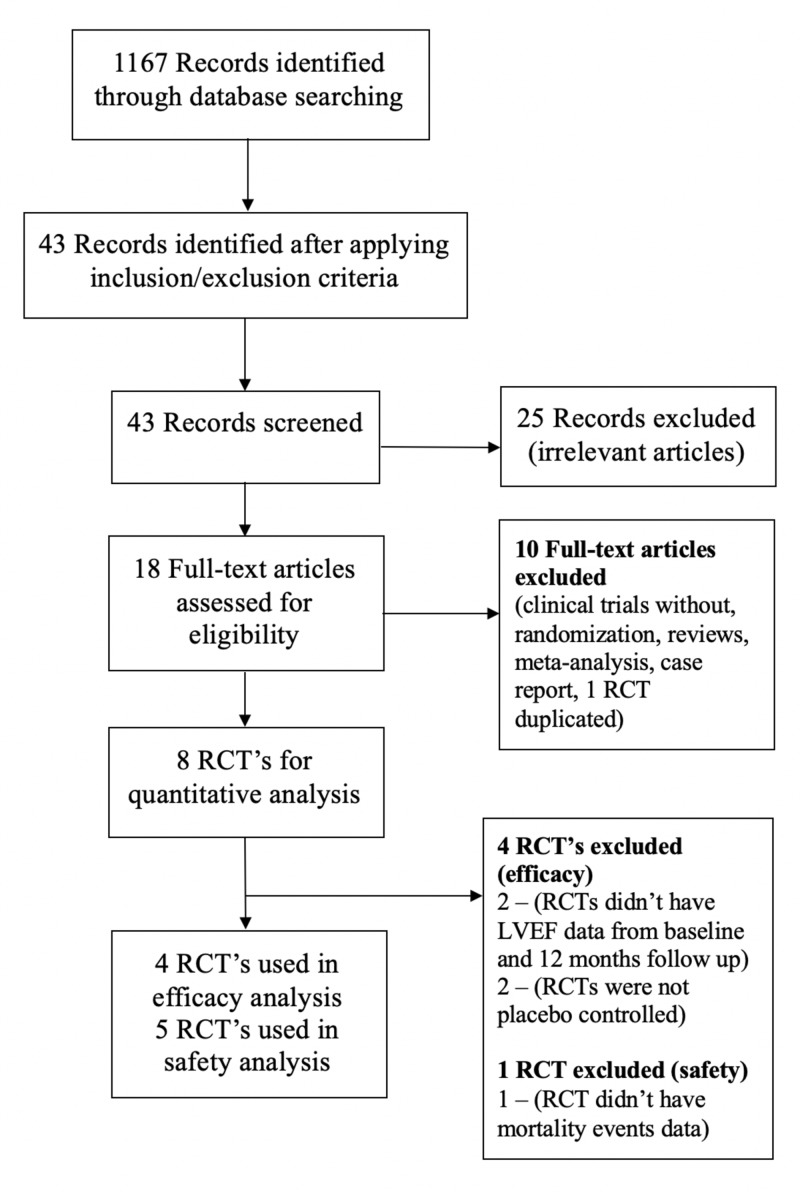
Summary of study flow (PRISMA flow diagram) RCT= Randomized Controlled Trial; LVEF = Left Ventricular Ejection Fraction; PRISMA = Preferred Reporting Items for Systematic Reviews and Meta-Analyses

The search was conducted through the PubMed search engine between 2017 and 2019 using the following keywords as illustrated in Table 1. This search revealed 1167 published, peer-reviewed scientific articles. Forty-three (43) of the 1167 scientific papers met the inclusion/exclusion criterion. A total of 18 initial articles were obtained from analyzing the titles and abstracts of the 43 search results, which included one meta-analysis, two review articles, eight RCTs, six non-randomized studies, and one case report. All the data were collected ethically and legally. This is summarized as the flow of search trial illustrated by preferred reporting items for systematic reviews and meta-analyses (PRISMA) flow diagram (Figure 1) [12]. The basal characteristics of the selected trials are summarized in Tables 2-3.

**Table 1:** Search results from PubMed

	Hits	Cumulative Hits	Combined Cumulative Hits
heart failure	240477	((((((heart failure) OR "systolic heart failure") OR cardiomyopathies) OR "ischemic cardiomyopathy") OR "nonischemic cardiomyopathy") OR "chronic ischemic heart disease") OR "cardiomyopathy, dilated" = 316842	((((((((((heart failure) OR "systolic heart failure") OR cardiomyopathies) OR "ischemic cardiomyopathy") OR "nonischemic cardiomyopathy") OR "chronic ischemic heart disease") OR "cardiomyopathy, dilated")) AND (((((autologous adult bone marrow-derived stem cells) OR mesenchymal stem cells) OR stem cell therapy) OR human induced pluripotent stem cells) OR "injection of stem cells")) AND heart failure therapy) AND (((((safety) OR efficacy) OR effectiveness) OR prognosis) OR role) = 1167 Forty three of the 1167 scientific papers met the inclusion/exclusion criterion
“systolic heart failure”	2736		
cardiomyopathies	92422		
“ischemic cardiomyopathy”	3604		
“nonischemic cardiomyopathy”	905		
“chronic ischemic heart disease”	889		
“cardiomyopathy, dilated”	15207		
stem cell therapy	175368	((((autologous adult bone marrow-derived stem cells) OR mesenchymal stem cells) OR stem cell therapy) OR human induced pluripotent stem cells) OR "injection of stem cells" = 220626	
autologous adult bone marrow-derived stem cells	823		
mesenchymal stem cells	57813		
human-induced pluripotent stem cells	16670		
“injection of stem cells”	15827		
heart failure therapy	149015	149015	
safety	578532	((((safety) OR efficacy) OR effectiveness) OR prognosis) OR role = 5217604	
efficacy	752718		
effectiveness	424342		
role	2511565		
prognosis	1714242		

**Table 2:** Basal characteristics of selected RCTs included in this meta-analysis Abbreviations: T = Treatment / C = Control Arm; Ischemia-Tolerant MSCs = itMSCs; Ischemic Cardiomyopathy = ICM; Cardiac Magnetic Resonance = CMR; Heart failure with Reduced Ejection Fraction = HFrEF; New York Heart Association = NYHA; INTRAVENOUS = IV; Umbilical Cord–Derived Mesenchymal Stem Cells = UC-MSC; 6 Minute Walk Test = 6MWT; Left Ventricular End Diastolic Volume = LVEDV; Left Ventricular End Diastolic Diameter = LVEDD; Non-Ischemic Dilated CardioMyopathy = NIDCM; Mesenchymal Precursor Cells = MPCs; Left Ventricular Assist Device= LVAD; Bridge to Therapy = BT; Destination Therapy = DT; Echocardiography = ECHO; Coronary Artery Bypass Grafting= CABG; Bone Marrow Mononuclear Cells= BMMNC; Left Ventricular = LV; Myocardial Infarction = MI; Heart Failure = HF; Congestive Heart Failure = CHF; Bone Marrow Mesenchymal Stem Cells = BM-MSCs; Percutaneous Coronary Intervention = PCI; Anterior Myocardial Infarction = AMI; Left Anterior Descending = LAD; Ejection Fraction = EF; Electrocardiogram = EKG; Single Photon Emission Computed Tomography = SPECT; Human Mesenchymal Stem Cell = hMSCs; Cardiovascular = CV; Bone Marrow = BM; Left Ventricular Ejection Fraction = LVEF; Ischemic Heart Disease = IHD; Cardiac Computed Tomography = Cardiac CT

SI #	Author/Year	Objective	Study type	Rationale	Sample size (T vs C)	Mean Age, (Yr.) (T vs C)	Gender, % (T vs C)	Trial inclusion	Assessment
Randomized Placebo-Controlled Parallel Clinical Trials									
1.	Yau TM et al., 2019 [13]	Efficacy & adverse effects of MPCs during LVAD implant	Randomized, Placebo phase 2 clinical trial	MPCs may suppress inflammatory cytokines that cause infections, bleeding and thrombosis	159 patients (106 vs 53)	55.5 vs 56.9	11.3% vs 11.3% women	end-stage heart failure for a clinically indicated LVAD for BT or DT	ECHO 6MWT
2.	Qi Z et al., 2018 [14]	Effectiveness of isolated CABG combined with BMMNC delivered via graft vessels to improve LV dyssynchrony	Randomized placebo-controlled trial	BMMNC via graft vessels to improve LV dyssynchrony in patients with previous MI and chronic HF.	42 patients (24 vs 18)	57.8﻿±8.5 vs 56.5±9	95.8% vs 94.4% men	18-75 years with CHF and suitable for elective CABG surgery	ECHO
3.	﻿Kim SH et al., 2018 [15]	Safety & efficacy of autologous BM-MSCs at 1-month post (PCI) in anterior MI	Randomized placebo-controlled trial	Studies have shown that cardiac transfer of unfractionated BM-MSCs and progenitor cells enhance functional recovery after AMI	26 patients (14 vs 12)	55.3±8.6 vs 57.8±8.9	100% vs 100% men	<72h after successful revascularization of anterior AMI (residual stenosis <30% of LAD artery infarction) and EF ≤ 40%	EKG gated SPECT ECHO
4.	Chart 1 Trial Design - Bartunek J et al., 2016 [16] Results at 39 weeks - Bartunek J et al., 2017 [17] Post Hoc analysis @ 52 weeks -Teerlink JR et al., 2017 [18]	﻿impact of the intramyocardial administration of BM-derived, lineage directed autologous cardiopoietic MSC’s on LV remodeling in patients with advanced HF enrolled in the CHART-1 study	﻿Multinational, randomized, double-blind, sham-controlled study	﻿C3BS-CQR-1 is a cardiopoiesis guided preparation of patient-derived MSC’s that has been proposed to potentially improve symptoms, functional capacity, and clinical outcomes in patients with advanced HF ﻿Guided	271 patients (120 vs 151) for efficacy analysis (120 vs 170) for safety analysis	61.6±8.6 vs 62.1±8.7	89.2% vs 90.1% men	﻿symptomatic advanced HF secondary to IHD, and reduced LVEF <35% BY JOHN	ECHO
5.	Bartolucci J et al., 2017 [19]	﻿Safety & efficacy of IV infusion of UC-MSC in patients with chronic stable HF and reduced EF	Prospective, randomized, double-blinded placebo-controlled trial	﻿UC-MSC are easily accessible and expanded in vitro, possess distinct properties, and improve myocardial remodeling and function in experimental models of CV disease	30 patients (15 vs 15)	﻿57.3±10 vs 57.2±11.6	80% vs 93.3% men	Chronic HFrEF with NYHA classification I to III and LVEF ≤40%	ECHO CMR
Randomized Placebo-Controlled Crossover Clinical Trials									
6.	(Rimecard trial) Butler J et al., 2017 [20]	﻿Safety & efficacy of IV administered itMSCs in patients with non-ICM	Single-blind, placebo-controlled, crossover, randomized phase II-a trial	﻿Benefits of MSC therapy in HF may be related to paracrine properties and anti-inflammatory activities.	22 patients (10 vs 12)	﻿47.3 vs 47.3	﻿59.1% vs 59.1% men	﻿Non-ICM patients with LVEF ≤40% and absent hyperenhancement on CMR imaging	ECHO
Randomized Controlled Clinical Trials Using Stem Cell Therapy Itself as Control									
7.	(POSEIDON-DCM Trial ) Hare JM et al., 2017 [21]	﻿Safety & efficacy of autologous (auto) vs. allogeneic (allo) BM-derived hMSC’s in NIDCM	Randomized Phase I/II Pilot Study	﻿hMSCs exert antifibrotic and pro-regenerative effects leading to improved ventricular function and architecture in antecedent MI. As MSCs have anti-inflammatory effects and stimulate restoration of endothelial health	37 patients (16 vs 18)	54.4 vs 57.4	77.8% vs 62.5% men	﻿NIDCM with an EF <40% and either an LVEDD >5.9 cm in male and >5.6 cm in female or an LVEDV index > 125 ml/m2, as previously described	ECHO 6MWT
8.	(TRIDENT Trial) ﻿Florea V et al., 2017 [22]	﻿Safety & efficacy of two doses of allogeneic BM-derived hMSC identically delivered in patients with ICM	Phase II, Randomized, Blinded, Study	﻿Cell dose and concentration play crucial roles in phenotypic responses to cell-based therapy for heart failure	30 Patients (15 vs 15)	65.6±9 vs 66.8±12	100% vs 80% men	chronic ischemic LV dysfunction secondary to MI on maximal appropriate medical therapy with a confirmed EF ≤ 50%	ECHO CARDIAC CT

**Table 3:** Stem cell dose and route of administration used in the selected RCTs Abbreviations: Mesenchymal Precursor Cells = MPC; Bone Marrow Mononuclear Cells = BM-MNC; Bone Marrow Mesenchymal Stem Cells = BM-MSC; Mesenchymal Stem Cells = MSC; Human Mesenchymal Stem Cells = hMSCs; Umbilical Cord–Derived Mesenchymal Stem Cells = UC-MSC; Allogeneic Bone Marrow-Derived Human Mesenchymal Stem Cells = BM-hMSCs; Ischemia-Tolerant ﻿Mesenchymal Stem Cell = itMSCs; RCA = Right Coronary Artery; LCX = Left Circumflex coronary artery; RCT = Randomized Controlled Trial

SI #	Route of administration	Stem cell dose
1.	Intramyocardial injections (IM)	Allogeneic MPCs = 150 million
2.	﻿Injected via the saphenous vein bypass graft after distal anastomosis of the RCA and LCX	BMMNC’s ﻿= 10^6^/mL
3.	Intracoronary delivery	﻿Autologous BM-MSC = 7.2 ± 0.90 × 10^7^ cells
4.	Intramyocardial injections (IM)	﻿﻿MSC Cardiopoietic cells = 57–60x10^6^ cells/mL
5.	﻿Intravenous infusion (IV)	﻿Allogenic UC-MSCs = 1×10^6^cells/kg
6.	Intravenous infusion (IV)	itMSCs = 1.5×10^6^ cells/kg
7.	Trans endocardial injections (TESI)	﻿Allologous-hMSCs or Autologous-hMSCs = 100 million
8.	Trans endocardial injections (TESI)	﻿allogeneic BM-hMSCs = (20 million x n=15) or (100 million x n=15)

Selected studies with characteristics

According to the eligibility criteria, eight RCTs were potentially eligible for inclusion in this meta-analysis. From the first collection, three RCTs were excluded from the safety analysis, and four were excluded from the efficacy analysis. This meta-analysis included 289 patients who underwent stem cell transplantation for advanced heart failure, with 280 patients as controls. Few studies were excluded from the meta-analysis either because the studies were inappropriate or because they lacked the necessary data or because they were duplicated RCTs as clearly illustrated in the PRISMA flow diagram [12]. Here, the duplicated trials mean that the same RCT with additional follow-up data at different time points (e.g. reports at 39 weeks and 52 weeks) were published as a separate article. Finally, five RCTs were included for the safety analysis [13,15-20] and four RCTs were included for the efficacy analysis [14-19] in this meta-analysis. The efficacy analysis of this meta-analysis includes 172 patients who underwent stem cell transplantation for advanced heart failure, with 195 patients as controls. The safety analysis of this meta-analysis includes 265 patients who underwent stem cell transplantation for advanced heart failure, with 261 patients as controls. Two studies that were not included in both the safety and efficacy analyses, as they were inappropriate, are described hereunder; one was by Hare JM et al. [21], in which autologous vs. allogeneic bone marrow-derived mesenchymal stem cells were administered via the trans-endocardial route for advanced heart failure and an efficacy analysis comparing two cell sources was reported, and the other trial was by Florea V et al. [22], in which 100 vs. 20 million allogeneic bone marrow-derived mesenchymal stem cells were administered via the trans-endocardial route for advanced heart failure and an efficacy analysis comparing two cell doses was reported. We excluded these two trials from our analysis, as they were not placebo-controlled.

Quality assessment

**Figure 2:**
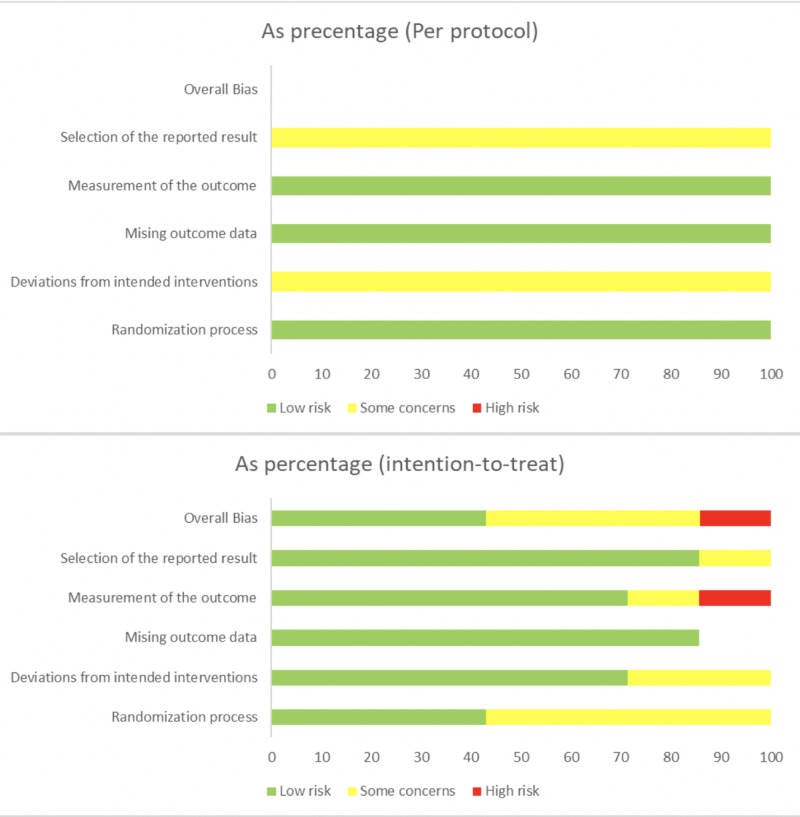
Illustrative summary of bias of the selected RCTs based on the Cochrane Risk of Bias (RoB) 2 tool RCT = Randomized Controlled Trial

The quality assessment data of these eight RCTs are described below (Table 4). All the eight trials were randomized, but only four of them described clearly the method used to generate randomized sequences. Seven trials clearly describe blinding the participants, but only six trials clearly describe blinding the investigators. Five trials reported clearly about blinding outcome assessors, and one trial did not blind outcome assessors. All the eight trials reported adequate details about the loss of participant follow-up. The summary of the risk of bias for the eight RCTs is shown illustratively using the RoB tool [7] (Figure 2). During the quality assessment, any discrepancy between the authors was resolved by consensus. Seven of the eight studies were shown to have either low risk of bias or some concerns while one study was shown to have a high risk of bias.

**Table 4:** Quality assessment data collected from the selected RCTs RCT = Randomized Controlled Trial

Study	Randomization sequence method	Allocation concealment method	Baseline characteristics similarity	Blinding of Outcome assessors	Blinding of Investigators and participants	Appropriate pre-specified outcome analysis method	Was outcome data available for all participants	Early RCT Discontinuation	Were all assigned patients treated
Yau TM et al., 2019 [13]	yes	yes	yes	yes	yes	yes	almost	no	yes
Qi Z et al., 2018 [14]	yes	yes	yes	yes	yes	unclear	yes	no	yes
Kim SH et al., 2018 [15]	possibly yes	unclear	yes	unclear	yes	yes	almost	no	yes
Bartunek J et al., 2017 [17]	yes	yes	yes	yes	yes	yes	no	no	almost
Bartolucci J et al., 2017 [19]	possibly yes	unclear	yes	yes	yes	yes	almost	no	almost
Butler J et al., 2017 [20]	possibly yes	unclear	yes	no	Participants only	yes	yes	no	yes
Hare JM et al., 2017 [21]	possibly yes	unclear	yes	unclear	unclear	yes	yes	no	almost
Florea V et al., 2017 [22]	yes	yes	no	yes	yes	yes	yes	no	yes

Efficacy analysis

**Figure 3:**
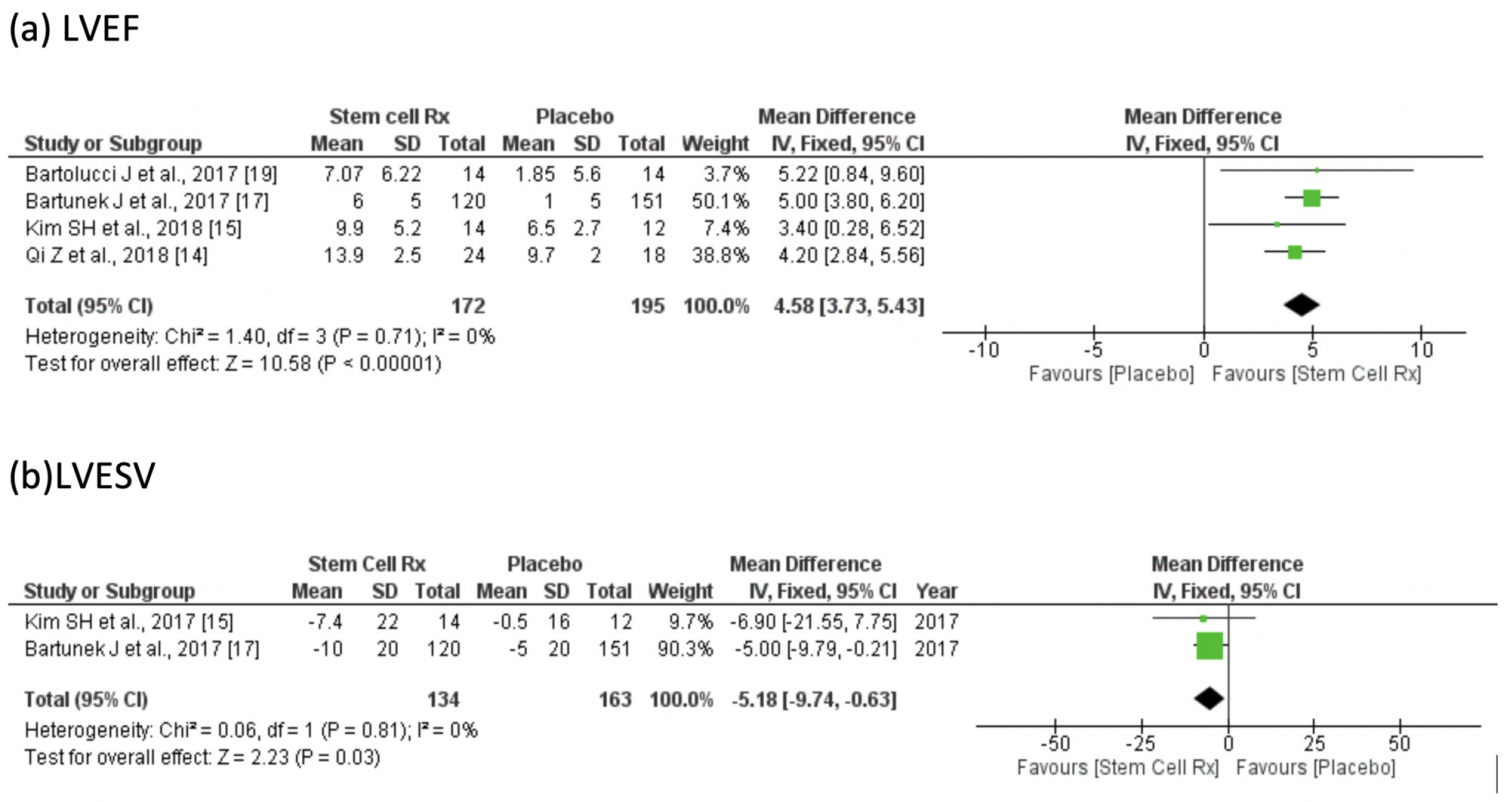
Forest plot of weighted mean difference (WMD), with a 95% confidence interval (CI) in (a) LVEF; (b) LVESV LVEF = Left Ventricular Ejection Fraction; LVESV = Left Ventricular End Systolic Volume

In the assessment of the efficacy of stem cell therapy vs placebo, stem cell therapy significantly improved LVEF by 4.58% (95% CI: 3.73-5.43%; p = 0.00001), and improved left ventricular end-systolic volume (LVESV) by -5.18 ml (95% CI: -9.74 to -0.63 ml; p =0.01). A forest plot illustrating the same is shown below (Figures 3a-3b). In the sensitivity analysis, LVEF improvement of stem cells as compared to placebo was not significantly affected by excluding data from any one of the included RCTs.

Mortality and safety analysis

**Figure 4:**
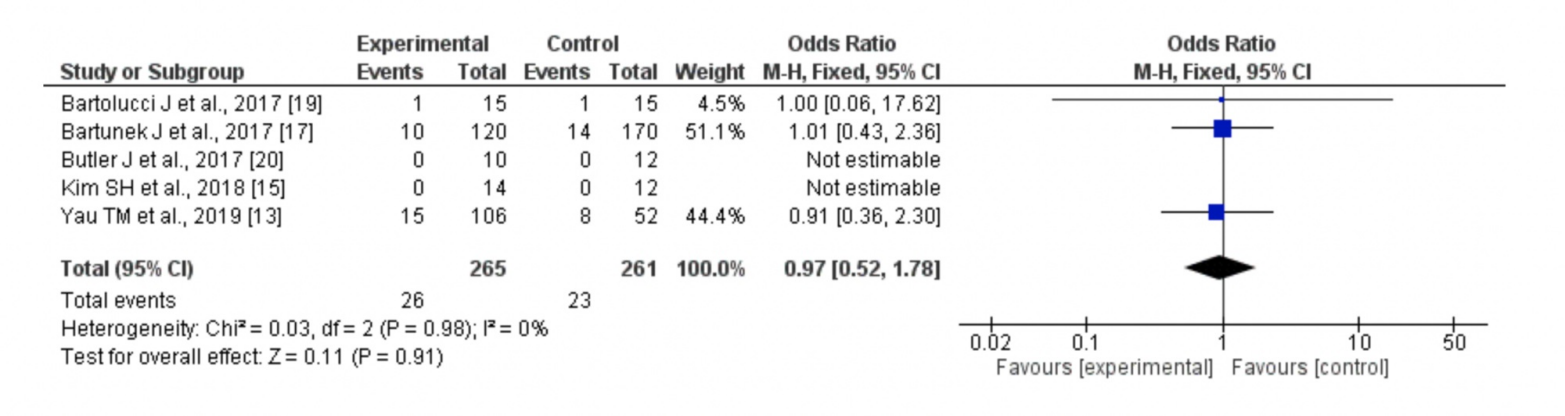
Forest plot of odds ratio (OR), with a 95% confidence interval (CI) on events of death in advanced heart failure patients treated with stem cell therapy compared with placebo

All-cause death occurred in 26 (9.8%) patients randomized to stem cell therapy as compared with 23 (8.8%) patients allocated as controls. No difference was observed in risk for all-cause death (OR 0.97; 95% CI: 0.52 to 1.78%; p = 0.91) between the stem cell and control groups (Figure 4). Serious cardiovascular events reported included death from cardiovascular disease, sudden cardiac death, arrhythmia, and myocardial infarction. Death from cardiovascular disease and sudden cardiac death were distributed among the stem cells and control groups. Most trials reported no or did not report procedure-related complications such as arrhythmia, stroke, and myocardial infarction. These results correlate well with the previous meta-analysis conducted in 2016 [23].

## Discussion

This meta-analysis was conducted to study and quantify the efficacy and safety of stem cell therapy in advanced heart failure, and it indicates that the use of stem cell therapy in advanced heart failure is safe. It also shows that there might be moderate improvement in LVEF and cardiac volumes (LVESV) in advanced heart failure with the application of stem cell therapy. The results section gives statistical proof supporting the above statements. The most important question that comes to our mind before we can proceed with stem cell research is "how does stem cell therapy work on the damaged myocardium?" The mechanism of action of stem cell therapy is debatable. The mechanism commonly attributed to stem cell therapy is the regeneration of heart cells, leading to a restored, functioning myocardium. However, the immediate cardioprotective effects of stem cells within one day of administration questions this line of thought [24]. These facts conclude that the effects of stem cells cannot be solely due to regeneration. Thus, these effects could be due to some paracrine effects of stem cells, such as increasing cell proliferation, stimulated cell recovery, apoptosis prevention, and promoting healing [25-28]. As paracrine effects can improve over time, a longer follow-up is essential after stem cell transplantation. One of the prime sources of stem cells is bone marrow. Stem cells derived from bone marrow have their subpopulations such as multipotent adult progenitor cells (MAPCs), endothelial progenitor cells (EPCs), hematopoietic stem cells (HSCs), and mesenchymal stem cells (MSCs) [29]. Therefore, there could be a combination of multiple paracrine effects from each of these different cell types in the bone marrow. Thus, the likely mechanism involved could be a combination of multiple mechanisms. There are many other sources for stem cells, such as tissue-derived, embryonic-derived, and reprogrammed cells, which are still under investigation. Also, there are many routes of administration of stem cells such as intracoronary, intravenous infusion, epicardial, or endocardial injection via a catheter (Intramyocardial) [29]. Among these, there is some evidence showing better stem cell retention with intramyocardial (10%) when compared to intracoronary (3%) [30]. These facts emphasize the need for a more structured RCT with cluster groups comparing the different stem cell sources and routes of administration. Also, the use of higher doses of stem cell therapy or allogenic type of stem cell therapy seems to be more effective in improving LVEF as compared to lower doses or autologous stem cell sources, as indicated by the POSEIDON (Prevention of Contrast Renal Injury with Different Hydration Strategies) trial and TRIDENT (Triple Therapy Prevention of Recurrent Intracerebral Disease Events) trial [21-22]. Together, therapeutic applications, such as higher doses and allogenic sources of stem cell therapy, may contribute to a better outcome when applying stem cell therapy in advanced heart failure.

Relevant RCTs included in this meta-analysis

Refer to Table 2 for basal characteristics.

Study #1 (Yau TM et al., 2019 [13]) is a randomized-placebo controlled phase 2 clinical trial evaluating the efficacy and adverse effects of MPCs during a left ventricular assist device (LVAD) implant. This was assessed through two primary endpoints: (1) the primary efficacy endpoint was the proportion of successful temporary weaning of LVAD support (of three planned assessments) within six months of randomization, (2) the primary safety endpoint was the incidence of adverse events related to the intervention such as myocarditis, myocardial rupture, and immune and hypersensitivity reactions. The results of the study were: (1) the mean proportion of successful temporary weaning from LVAD support was 61% in the MPC group and 58% in the control group, which was below the predefined threshold for success, and (2) no patients experienced a primary safety endpoint. Thus, the resulting conclusion was that intramyocardial injections of MPCs compared with the sham treatment did not improve successful temporary weaning of LVAD support, but the use of MPCs was safe. The results of this study were limited due to the following reasons: (1) at efficacy endpoints such as ECHO, functional status may not equally apply to patients receiving mechanical circulatory support in traditional heart failure trials, (2) signal of treatment might be reduced in relatively small trials, when there is a wide spectrum of patients (which was done in an effort to increase generalizability), and (3) international normalized ratio values or platelet counts were not collected in a systematic manner. However, since non-gastrointestinal tract bleeding events did not differ between the two groups, reduced anticoagulation cannot be attributed as the sole cause of reduced gastrointestinal bleeds [13].

Study #2 (Qi Z et al., 2018 [14]) is a randomized, placebo-controlled trial evaluating the efficacy of combining coronary artery bypass graft (CABG) with bone marrow mononuclear cells (BMMNC) as compared to CABG with placebo administration to improve left ventricular (LV) dyssynchrony. This was assessed by measuring the difference in time-to-peak radial strain between the earliest and the latest activated segments on LV short-axis images at the apical (RSTa), at the mitral annulus (RSTb), and at the papillary muscle (RSTm) level through ﻿2D strain imaging. The results of the study were (1) the LV dyssynchrony rate was improved with CABG + BMMNC when compared to the CABG only group, and (2) the LV synchrony deterioration rate in the CABG + BMMNC was significantly lower when compared to the CABG only group. Thus, the resulting conclusion was a better improvement of left ventricular dyssynchrony when combining CABG with BMMNC than in CABG only. The results of this study were limited due to the small sample size and, hence, this trial is to be considered as a pilot study and, furthermore, extensive multicenter studies should be considered [14].

Study #3 (Kim SH et al., 2018 [15]) is a randomized, placebo-controlled trial evaluating the safety and efficacy of autologous bone marrow mesenchymal stem cells (BM-MSCs) at one-month post-percutaneous coronary intervention (PCI) in anterior MI. This was assessed through the primary endpoint in which the change in LVEF at four months follow-up as compared to the baseline. The results of the study were (1) the global LVEF was ﻿33.6 ± 4.7% in the BM-MSC group and﻿ 35.4 ± 3.0% in the control group, (2) the global LVEF increased ﻿by 8.8 ± 2.9% in the BM-MSC group and ﻿4.8 ± 1.9% in the control group after four months, (3) the risk of adverse clinical events, proarrhythmic effects, and in-stent restenosis was not increased by cell transfer, and (4) the LVEF was significantly increased at the fourth month and twelfth month follow-ups when compared to the baseline in the BM-MSC group, but this was not seen in the control group. Thus, the resulting conclusion was intracoronary injections of autologous BM-MSCs when compared with the sham treatment was safe, and it also improved the LVEF at four months (SPECT/ECHO) and 12 months (ECHO) follow-up in anterior acute myocardial infarction (AMI) patients. The results of this study were limited due to the following reasons: (1) small sample size, (2) data were collected at an earlier time point, and (3) methods of left ventricular functional assessment were limited [15].

Study #4 (Bartunek J et al., 2017 [17]) is a multinational, randomized, double-blind, sham-controlled study aimed to validate cardiopoiesis-based biotherapy in a larger heart-failure cohort. This was assessed by the following endpoints: the primary safety endpoint was assessed by SAEs and primary efficacy endpoint was assessed by left ventricular end-diastolic volume (LVEDV), ejection fraction (EF), and the six-min walk test (6MWT) at 39 weeks and later at 52 weeks was assessed by ﻿a change from the baseline in both LVEDV and LVESV. The results of the study at 39 weeks were: (1) the primary outcome was neutral, (2) the only benefit of cell treatment was noticed in patients with baseline LVEDV 200-370 ml, and (3) no difference in serious adverse events (SAEs). At 52 weeks, the results were (1) both LVEDV and LVESV significantly decreased following C3BS-CQR-1 therapy, (2) there was a reduction of 17 and 12.8 ml of LVEDV and LVESV, respectively, at one year, and (3) the results remained consistent after adjusting for multiple variables. Thus, the resulting conclusion at 39 weeks is that the primary endpoint was neutral and further evaluation was needed for cell therapy in elevated LVEDV and later at 52 weeks, the resulting conclusion was (1) significant reverse remodeling noticed after cardiopoietic cells (C3BS-CQR-1) therapy as shown by progressive decrease in both LVEDV and LVESV, which was compared among the two arms of the study. The results of this study were limited at 39 weeks due to the following reasons: (1) study required a longer follow-up, (2) the result was biased by a modified intent to treat set, (3) the study population was predominantly Caucasian men and limited at 52 weeks due to the following reasons: (1) all conclusions are subject to confirmation, since it is a post-hoc analysis of the Chart 1 study, (2) treatment effects based on the number of injections lacks comparison as the controls were not given any injections, and (3) sample size is small, raising the possibility of play of chance [16-18].

Study #5 (Bartolucci J et al., 2017 [19]) is a prospective, randomized, double-blinded placebo-controlled trial evaluating the safety and efficacy of umbilical cord mesenchymal stem cells (UC-MSC) intravenous (IV) infusion in patients with chronic stable heart failure (HF) and reduced ejection fraction (EF). This was assessed by ﻿the following endpoints, safety endpoint was assessed by SAEs, adverse events (AEs), major adverse cardiovascular events (MACEs), and primary efficacy endpoints were assessed by change in LVEF, LVESV, and LVEDV based on ECHO and cardiac magnetic resonance (CMR). The results of the study were: (1) no adverse events noticed in UC-MSC-treated patients, (2) UC-MSC treated group showed significant improvements in LVEF at three, six, and 12 months assessed by ECHO and CMR, and (3) all follow-up patients of the UC-MSC group showed improvements in the hearts' function based on New York Health Association (NYHA). Thus, the resulting conclusion was that the intravenous infusion of UC-MSC was safe and improved LV function, functional status, and quality of life in heart failure with reduced ejection fraction (HFrEF). The results of this study were limited due to the following reasons: (1) a small number of participants in each patient group, and (2) myocardial perfusion and fibrosis measurements could not be done due to non-contrast CMR and software restraints [19].

Study #6 (Butler J et al., 2017 [20]) is a single-blind, placebo-controlled, crossover, randomized phase II-a clinical trial evaluating the safety and efficacy of IV-administered ischemia tolerant mesenchymal stem cells (itMSCs) in patients with non-ischemic cardiomyopathy (ICM). This was assessed by ﻿the following endpoints: (1) the safety endpoint was assessed by SAEs, all-cause mortality, and all-cause hospitalization at days 30, 60, 90, 120, 150, 180, 270, and 450 post-initial infusion, and (2) the primary efficacy endpoint was assessed by a change in LVEF, scar size, NYHA, and pro-BNP. The results of the study were: (1) no significant difference in SAEs, death, or hospitalization, (2) change in LVEF, LVEDV, and LVESV was similar in both groups, and (3) 6MWT, functional status scores increased with the itMSC group. Thus, the resulting conclusion was that itMSC therapy is safe, with improved functional capacity and health status. The results of this study were limited due to the following reasons: (1) cannot determine if the adverse effect at >90 days was due to the placebo effect, a delayed consequence of cell therapy, or random chance, and (2) it was conducted by cells grown under chronic hypoxia and not compared with cells grown in normoxic conditions [20].

Study #7 (﻿Hare JM et al., 2017 [21]) is a randomized phase I/II pilot study evaluating the safety and efficacy of autologous (auto) vs. allogeneic (allo) BM-derived human mesenchymal stem cell (hMSCs) in non-ischemic dilated cardiomyopathy (NIDCM). This was assessed by ﻿the following endpoints, safety endpoint was assessed by SAEs, AEs, and MACEs, and the primary efficacy endpoint was assessed by the LV structure and function, NYHA, 6MWT, Minnesota living with heart failure questionnaire (MLHFQ). The results of the study were (1) the 12-month SAE result was 28.2% in allo vs. 63.5% in auto hMSCs, (2) EF increased in allo by 8 vs in auto by 5.4, (3) six MWT was increased by 37 meters for allo but not for auto, (4) MLHFQ decreased both in allo and in auto, (5) the MACE rate lower in allo when compared to auto. Thus, the resulting conclusion was that there was more excellent safety and clinically meaningful efficacy in allo-hMSCs when compared to auto-hMSCs in NIDCM patients. The results of this study were limited due to the following reasons: (1) the study lacked a placebo group, (2) loss of patient due to the withdrawal of consent and follow-up, and (3) small sample size [21].

Study #8 (Florea V et al., 2017 [22]) is a phase II, randomized, blinded clinical trial evaluating the safety and efficacy of two different doses of allogeneic BM-derived hMSC identically delivered in patients with ICM. This was assessed by ﻿the following endpoints: the safety endpoint was assessed by SAEs at one month; AEs, SAEs at six and 12 months; MACE and re-hospitalization, the primary efficacy endpoint was assessed by a change in LVEF, scar size, NYHA, and pro-BNP. The results of the study were: (1) no adverse SAEs at 30 days or 12 months, (2) MACE rate was 20% in 20M and 13.3% in 100M, (3) worsening HF-induced re-hospitalization was 20% in 20M and 7.1% in 100M, (4) scar size reduced similarly in both groups, (5) EF improved only in the 100M group by 3.7U, (6) NYHA status improved at 12 m in 35.7% patients receiving 20M and 42.9% in patients receiving 100M, (7) pro-BNP increased at 12 m in the 20M patient group but not in the 100M patient group. Thus, the resulting conclusion was that 100 million patient groups had an improvement in EF, but both 20 million and 100 million patient groups had a reduction in scar size, and thus 100 million doses of allogeneic BM-derived hMSC is significantly better than a 20-million dose of allogeneic bone marrow-derived hMSC. The results of this study were limited due to the following reasons: (1) a small number of participants per group, and (2) lack of a placebo group [22].

Limitations of our meta-analysis

1. A relatively small sample size and a limited number of RCTs were included. The sample size further affected in some RCTs by the loss of study subjects either due to early death or other reasons.

2. There were some quality concerns with some of the RCTs included, especially one study was low in quality.

3. The moderate rise of LVEF in the heart failure population may be negligible.

4. Due to the few numbers of RCTs enrolled for this meta-analysis, the source of heterogeneity could not be explored with meta-regression. Thus, the interpretation of the actual intervention effect may be affected.

5. A literature search was conducted in a single electronic search engine (PubMed), so relevant articles listed in other search engines might have been missed, thus limiting the comprehensiveness of this meta-analysis.

6. No detailed subgroup analysis was performed due to the small sample size of the studies.

7. Studies published in other/non-English languages (except if there was a translated version readily available) have not been reviewed.

## Conclusions

The target of this study was to increase the curiosity of laymen and researchers to explore the world of stem cell therapy more. This meta-analysis of five RCTs for safety analysis and four RCTs for efficacy analysis can be summarized as follows. Stem cell therapy causes a moderate increase in LVEF; an improvement in the LVESV volume was also seen in some studies and there was no increase in all-cause mortality. One study that did not show any positive effect on the LVEF was limited due to a short study period, and this RCT was not included in the efficacy analysis of this meta-analysis, as the difference between the mean LVEF at baseline and at 12 months' follow-up was not available. These findings suggest stem cell therapy can be added safely to the routine standard of care for advanced HF, and it can potentially be an excellent add-on therapy to the current standard of care. However, we believe that the above evidence, without accounting for study limitations, is still inconclusive to answer which specific stem cell type, dosage, and route and time of administration in various scenarios of advanced HF is most effective. We believe future, large-scale, multiarmed, randomized, placebo-controlled clinical trials dealing with the combination approach of stem cell therapy will be able to unearth this answer in definitive detail. It can also help future scientists in exploring and implementing the best approach for stem cell therapy. As an added benefit, we may be able to understand the cost-effectiveness and possibly understand the exact mechanism of action involved in stem cell therapy. So, the questions left in our mind are, "which stem cell lineage to consider?", "at which route of administration", and "at what concentration?" As some trials have shown that the use of a higher dosage and concentration impacts the effect of stem cell therapy on heart tissue.

## References

[1] Zhu L, Yue A, Ruan Z, Yin Y, Wang R, Ren Y, Chen G (2017). Left ventricular improvement due to allogeneic CB-MNCs transplantation in a chronic heart failure six-years after myocardial infarction. Cardiol J.

[2] Mozaffarian D, Benjamin EJ, Go AS (2016). Heart Disease and Stroke Statistics—2016 Update. A report from the American Heart Association. Circulation.

[3] Heidenreich PA, Trogdon JG, Khavjou OA (2011). Forecasting the future of cardiovascular disease in the United States. A policy statement from the American Heart Association. Circulation.

[4] Williams AR, Hare JM, Dimmeler S, Losordo D (2011). Mesenchymal stem cells. Biology, pathophysiology, translational findings, and therapeutic implications for cardiac disease. Circ Res.

[5] Sanganalmath SK, Bolli R (2013). Cell therapy for heart failure. A comprehensive overview of experimental and clinical studies, current challenges, and future directions. Circ Res.

[6] Higgins JPT, Sterne JAC, Savović J A revised tool for assessing risk of bias in randomized trials. Cochrane Methods 2016.

[7] Shea BJ, Reeves BC, Wells G (2017). AMSTAR 2: a critical appraisal tool for systematic reviews that include randomised or non-randomised studies of healthcare interventions, or both. BMJ.

[8] Moola S, Munn Z, Tufanaru C (2017). Systematic reviews of etiology and risk. JBI Reviewer's Manual.

[9] Lockwood C, Munn Z, Porritt K (2015). Qualitative research synthesis: methodological guidance for systematic reviewers utilizing meta-aggregation. Int J Evid Based Healthc.

[10] McArthur A, Klugarova J, Yan H, Florescu S (2015). Innovations in the systematic review of text and opinion. Int J Evid Based Healthc.

[11] Tufanaru C, Munn Z, Aromataris E, Campbell J, Hopp L (2017). Systematic reviews of effectiveness. JBI Reviewer's Manual.

[12] Moher D, Liberati A, Tetzlaff J, Altman DG, PRISMA Group (2009). Preferred reporting items for systematic reviews and meta-analyses: the PRISMA statement. J Clin Epidemiol.

[13] Yau TM, Pagani FD, Mancini DM (2019). Intramyocardial injection of mesenchymal precursor cells and successful temporary weaning from left ventricular assist device support in patients with advanced heart failure. JAMA.

[14] Qi Z, Liu S, Duan F (2018). Effects of bone marrow mononuclear cells delivered through a graft vessel in patients with previous myocardial infarction and chronic heart failure: an echocardiographic study of left ventricular dyssynchrony. J Clin Ultrasound.

[15] Kim SH, Cho JH, Lee YH (2018). Improvement in left ventricular function with intracoronary mesenchymal stem cell therapy in a patient with anterior wall ST-segment elevation myocardial infarction. Cardiovasc Drugs Ther.

[16] Bartunek J, Davison B, Sherman W (2016). Congestive heart failure cardiopoietic regenerative therapy (CHART-1) trial design. Eur J Heart Fail.

[17] Bartunek J, Terzic A, Davison BA (2017). Cardiopoietic cell therapy for advanced ischaemic heart failure: results at 39 weeks of the prospective, randomized, double blind, sham-controlled CHART-1 clinical trial. Eur Heart J.

[18] Teerlink JR, Metra M, Filippatos GS (2017). Benefit of cardiopoietic mesenchymal stem cell therapy on left ventricular remodelling: results from the Congestive Heart Failure Cardiopoietic Regenerative Therapy (CHART-1) study. Eur J Heart Fail.

[19] Bartolucci J, Verdugo FJ, González PL (2017). Safety and efficacy of the intravenous infusion of umbilical cord mesenchymal stem cells in patients with heart failure. A phase 1/2 randomized controlled trial (RIMECARD trial [randomized clinical trial of intravenous infusion umbilical cord mesenchymal stem cells on cardiopathy]). Circ Res.

[20] Butler J, Epstein SE, Greene SJ (2017). Intravenous allogeneic mesenchymal stem cells for nonischemic cardiomyopathy. Safety and efficacy results of a phase II-A randomized trial. Circ Res.

[21] Hare JM, DiFede DL, Rieger AC (2017). Randomized comparison of allogeneic versus autologous mesenchymal stem cells for nonischemic dilated cardiomyopathy: POSEIDON-DCM trial. J Am Coll Cardiol.

[22] Florea V, Rieger AC, DiFede DL (2017). Dose comparison study of allogeneic mesenchymal stem cells in patients with ischemic cardiomyopathy (the TRIDENT study). Circ Res.

[23] Wen Y, Ding J, Zhang B, Gao Q (2018). Bone marrow-derived mononuclear cell therapy for nonischaemic dilated cardiomyopathy—a meta-analysis. Eur J Clin Invest.

[24] Kupatt C, Hinkel R, Lamparter M (2005). Retroinfusion of embryonic endothelial progenitor cells attenuates ischemia-reperfusion injury in pigs. Role of phosphatidylinositol 3-kinase/AKT kinase. Circulation.

[25] Kupatt C, Horstkotte J, Vlastos GA (2005). Embryonic endothelial progenitor cells expressing a broad range of proangiogenic and remodeling factors enhance vascularization and tissue recovery in acute and chronic ischemia. FASEB J.

[26] Gnecchi M, Zhang Z, Ni A, Dzau VJ (2008). Paracrine mechanisms in adult stem cell signaling and therapy. Circ Res.

[27] Penn MS, Dong F, Klein S, Mayorga M (2011). Stem cells for myocardial regeneration. Clin Pharmacol Ther.

[28] Uemura R, Xu M, Ahmad N, Ashraf M (2006). Bone marrow stem cells prevent left ventricular remodeling of ischemic heart through paracrine signaling. Circ Res.

[29] Jezierska-Woźniak K, Mystkowska D, Tutas A, Jurkowski MK (2011). Stem cells as therapy for cardiac disease — a review. Folia Histochem Cytobiol.

[30] Hou D, Youssef EA-S, Brinton TJ (2005). Radiolabeled cell distribution after intramyocardial, intracoronary, and interstitial retrograde coronary venous delivery. Implications for current clinical trials. Circulation.

